# Fossil amphibian offers insights into the interplay between monsoons and amphibian evolution in palaeoequatorial Late Triassic systems

**DOI:** 10.1098/rspb.2024.1041

**Published:** 2024-10-30

**Authors:** Calvin So, Aaron M. Kufner, Jason D. Pardo, Caian L. Edwards, Brandon R. Price, Joseph J. Bevitt, Amanda LeClair-Diaz, Lynette St. Clair, Josh Mann, Reba Teran, David M. Lovelace

**Affiliations:** ^1^ Department of Biological Sciences, George Washington University, Washington, DC, USA; ^2^ Department of Geoscience, University of Wisconsin-Madison, Madison, WI, USA; ^3^ University of Wisconsin Geology Museum, University of Wisconsin-Madison, Madison, WI, USA; ^4^ Negaunee Integrative Research Center, Field Museum of Natural History, Chicago, IL, USA; ^5^ University of Exeter, Exeter, UK; ^6^ Australian Centre for Neutron Scattering, Australian Nuclear Science and Technology Organisation, Lucas Heights, Sydney, New South Wales, Australia; ^7^ Fort Washakie Schools, Fremont County School District #21, 90 Ethete Road, Fort Washakie, WY 82514, USA; ^8^ Eastern Shoshone Tribal Historic Preservation Office, Building 17A, North Fork Road, Fort Washakie, WY 82514, USA

**Keywords:** Triassic, Temnospondyli, adaptation, estivation, megamonsoon

## Abstract

The severe greenhouse climate and seasonality of the early to mid-Late Triassic are thought to have limited terrestrial diversity at lower latitudes, but direct adaptations to these harsh conditions remain limited in vertebrates at the palaeoequator. Here, we present *Ninumbeehan dookoodukah* gen. et sp. nov., an early amphibian with specialized adaptations for seasonal estivation from the upper Jelm Formation of the Late Triassic of Wyoming, USA. *Ninumbeehan* are found in an association of vertebrate estivation burrows across a locally dense horizon, offering insights into the evolution and ecology of vertebrates amid the challenging conditions of low-latitude Late Triassic ecosystems. Estivation chambers were excavated within point bar deposits of an ephemeral river system, recording the cyclical signature of Triassic megamonsoons and documenting a vertebrate response to annual climate extremes across tens to hundreds of seasons. Phylogenetic analysis recovers *Ninumbeehan* within a group of temnospondyls characterized by fossorial adaptation, underscoring the widespread adoption of burrowing and estivation in total group Lissamphibia. *Ninumbeehan* hints at the pivotal role seasonal dynamics played in shaping amphibian evolution.

## 1. Introduction

The Triassic period (*ca* 252−201 Ma) was a critical interval in the evolution of terrestrial ecosystems; bookended by two of the five major biotic extinctions, the Triassic terrestrial biota records the origin of many modern terrestrial vertebrate groups at the zeniths of the Pangean supercontinent and the extremes of the Mesozoic greenhouse climate regime [1–3]. Low-latitude mid-Late Triassic strata record strong environmental fluctuations such as the waxing and waning of supercontinent-scale monsoonal systems (megamonsoons) that created extreme temperatures and seasonal climates in equatorial Pangea until a sudden departure from this regime in the mid-Carnian during the Carnian pluvial episode (CPE) [2,4–7]. Lethal daytime temperatures across equatorial latitudes and severe seasonal water stress in terrestrial ecosystems of the mid-Late Triassic are predicted by climate modelling [2,8,9], which may explain why palaeoequatorial vertebrate communities at this time differ in taxonomic composition from higher-latitude communities [10]. In particular, thermophysiological models have shown that daytime temperatures throughout this interval would have been lethal to some small-bodied taxa without adaptations for burrowing or estivation [11] and large-bodied taxa unable to shelter during daytime highs [12], which may explain the absence of large-bodied sauropodomorphs from Triassic equatorial faunas [10–12]. Given these extreme climatic conditions, we would expect to see direct evidence of physiological and behavioural adaptation to seasonal heat and water stress in equatorial vertebrate communities, such as excavation of burrows for estivation or shelter [11]. Estivation assemblages consisting of burrow casts that preserve their vertebrate tracemakers are common and readily identifiable in the earliest stages of Pangaea assembly [13]; burrow traces made by earliest Triassic therapsids are common at higher latitudes [14,15]. By contrast, evidence of vertebrate burrowing at the Triassic palaeoequator has been limited to ambiguous ichnofossils which may have been constructed by ‘lungfish’ [16,17] and rare associations with isolated putative stereospondyl tracemakers [18]. However, there remains little direct evidence of vertebrate behavioural adaptations to Late Triassic climate.

Here, we describe an unusually dense association of vertebrate estivation burrows from the early-Late Triassic (*ca* ≥231 Ma) upper Jelm Formation of Fremont County, Wyoming. Burrows contain well-preserved skeletal remains of a new species of stereospondyl amphibian with specialized adaptations for burrowing, unambiguously identifying these amphibians as the tracemakers. This represents direct evidence of estivation in the tropical Late Triassic, a behavioural (and implicitly physiological) adaptation to the early-Late Triassic megamonsoon.

## 2. Results

### (a) Geological setting

#### (i) Age constraints

**Figure 1.**
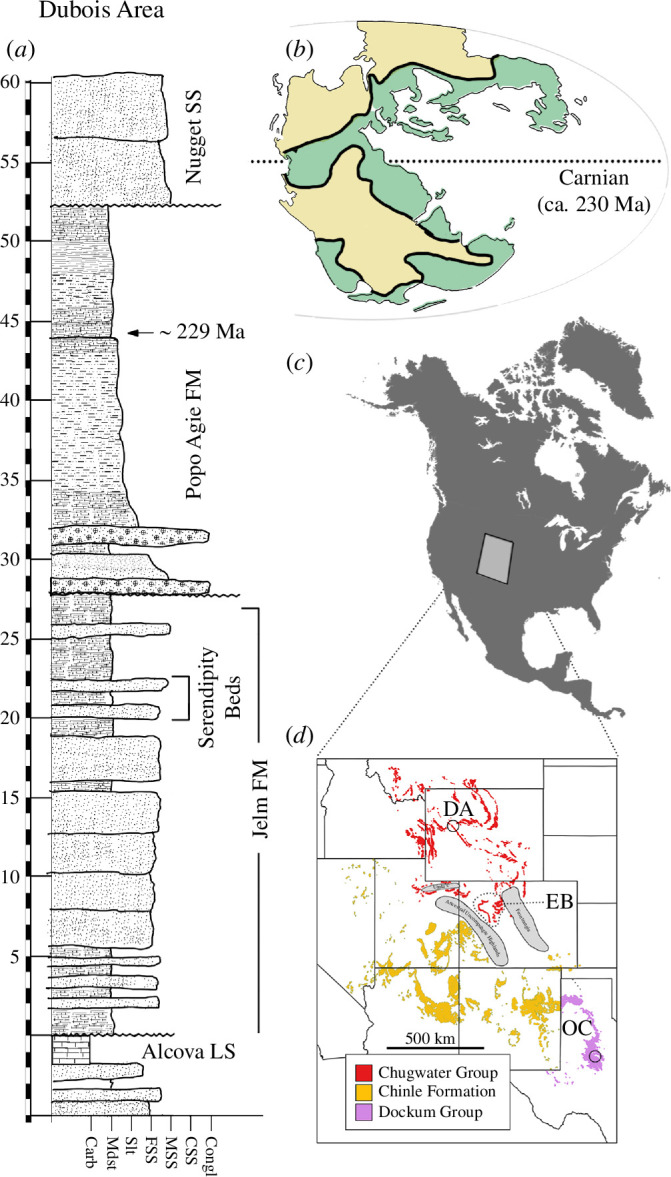
(*a*) Representative stratigraphic profile of the upper Chugwater Group in the Dubois Area modified after [19]. (*b*) Paleogeographic map showing Carnian precipitation (green area= *ca* 1-7 mm d^−1^) modified after [9]. (*c*) Modern geography of North America with grey square denoting position of (*d*). (*d*) demonstrates the stratigraphic areal distribution of continental Triassic units of the Rocky Mountain West, USA. Abbreviations: Carb, carbonate; Congl, conglomerate CSS, coarse-grained sandstone; DA, Dubois Area; EB, Eagle Basin; FM, Formation; FSS, fine-grained sandstone; LS, limestone; Mdst, mudstone; MSS, medium-grained sandstone; OC, Otis Chalk; Slt, siltstone; SS, sandstone.

In west-central Wyoming, the upper Chugwater Group consists of the Late Carnian-aged Popo Agie Formation (*ca* 231−227 Ma) and the underlying Jelm Formation (figure 1; [19–21]). There are no rigorous age constraints for the Jelm Formation. However, Lovelace *et al*. [19] used new fossil discoveries [22] and reported the first radioisotopic detrital ages that constrain the overlying Popo Agie Formation to the mid-Late Carnian (*ca* 231−227 Ma) [19] and the base of the Jelm Formation to no older than the earliest Anisian (*ca* 247 Ma) [23]. Given the stratigraphic position of the Serendipity beds in the uppermost Jelm, we infer this interval ranges from early-mid Carnian in age, pending more quantitative geochronology.

#### (ii) Burrows

**Figure 2.**
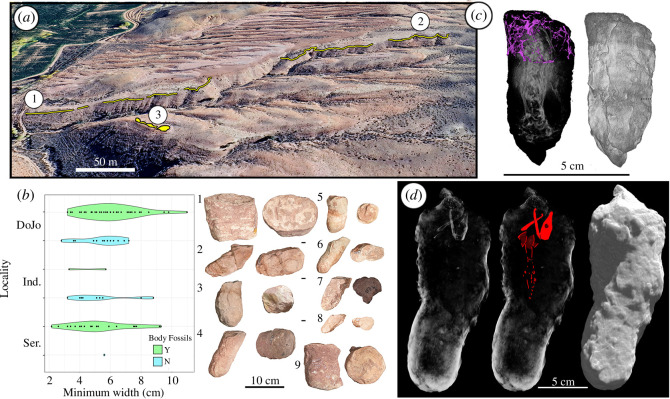
Burrow localities, size distribution, and morphotypes of the Serendipity beds (yellow lines), upper Jelm Formation, near Dubois, WY, and collection localities: (*a*) (1) Serendipity, (2) DoJo, and (3) Independence. (*b*) Plot of representative burrows (*n* = 88) from the three localities (data in the table 1) demonstrating size distribution and general morphology of burrows (1, UWGM 7191; 2, 7188; 3, 7193; 4, 7194; 5, 7196; 6, 7189; 7, 7192; 8, 7195; 9, 7190). (*c*) Neutron tomography rendering of UWGM 2204 (morphosource.org; ID:000609370) containing a skeleton and rhizoliths within burrow, purple = rhizolith network from top 2 cm of burrow (left), volume rendering (right). (*d*) Micro-computed tomography rendering of UWGM 7438 with a partially articulated skeleton (left), highlighted skeleton (middle), burrow cast volume rendering (right).

Vertebrate estivation burrows are present *ex situ* and *in situ* throughout exposures of the Serendipity beds. Burrows were surveyed across three collection areas: Serendipity, DoJo and Independence, recording the distribution of size, skeletal remains and burrow completeness (figure 2). Burrows are preserved as an infilled excavation (i.e. cast) that is lithologically similar but often readily separates from the host rock, which consists of laterally accreted sediment, suggestive of a point bar of a meandering river. Burrows exhibit a range of morphologies but are generally sub-vertical to vertical with no unambiguous bioglyphs, non-uniform diameter and, occasionally, a weakly pustulate surface (figure 2*b*). Burrow diameter corresponds closely to the skull width of the occupant. Most casts terminate at the base of the host sandstone and briefly penetrate into the underlying mudstones (electronic supplementary material, figure S1). Reduction haloes often encircle bone in the casts. Not all casts contain identifiable bone, and when bones are present, they are in varying states of articulation (e.g. figure 2*d*). The burrows are abundant and sometimes densely packed (electronic supplementary material, figure S1), and some adjacent burrows cross-cut one another. The abundance of burrow casts and the cross-cutting nature of some of those casts suggest recurrent episodes of excavation and time averaging of this assemblage.

**Table 1.** Select measurements of burrow casts from the Serendipity beds used in figure 2*b*. Minimum burrow diameter was measured at the truncated end of each burrow cast. The presence or absence of body fossils does not reflect whether remains were articulated or disarticulated.

Catalog Number	Site	Minimum Width of Truncated Top (cm)	Body Fossils Present?
UWGM 2204	Serendipity	3.2	Y
UWGM 2207	Serendipity	3.7	Y
UWGM 2208	Serendipity	4.1	Y
UWGM 5859	DoJo	6	Y
UWGM 5860	Serendipity	4.72	Y
UWGM 5863	Serendipity	5.9	Y
UWGM 5866	Serendipity	7.6	Y
UWGM 5868	Serendipity	5.9	Y
UWGM 5868	Serendipity	6.4	Y
UWGM 5870	DoJo	6	Y
UWGM 5875	DoJo	4.3	Y
UWGM 5879	Serendipity	2.16	Y
UWGM 5883	DoJo	8	Y
UWGM 5886	DoJo	5.8	Y
UWGM 5887	DoJo	6.5	Y
UWGM 5888	Serendipity	5.2	Y
UWGM 5890	DoJo	5.7	Y
UWGM 7187	Independence	4.1	N
UWGM 7188	Independence	5.3	N
UWGM 7189	Independence	3.2	N
UWGM 7190	Serendipity	9.3	Y
UWGM 7191	Independence	8.8	N
UWGM 7192	Independence	4	N
UWGM 7193	Independence	8	N
UWGM 7194	Independence	5.5	N
UWGM 7195	Independence	4.2	N
UWGM 7196	Independence	5.1	N
UWGM 7217	DoJo	5.57	Y
UWGM 7219	DoJo	9.5	Y
UWGM 7224	DoJo	5.41	Y
UWGM 7229	DoJo	4.75	Y
UWGM 7232	DoJo	3.4	Y
UWGM 7233	DoJo	3.5	Y
UWGM 7235	DoJo	4	Y
UWGM 7241	DoJo	7.2	Y
UWGM 7243	DoJo	6.4	Y
UWGM 7244	DoJo	6	Y
UWGM 7245	DoJo	7.1	Y
UWGM 7246	DoJo	6.2	N
UWGM 7247	DoJo	6.9	N
UWGM 7248	DoJo	7.2	N
UWGM 7249	DoJo	5.6	N
UWGM 7250	DoJo	6	N
UWGM 7251	DoJo	7.6	Y
UWGM 7252	DoJo	5.1	N
UWGM 7253	DoJo	3.7	N
UWGM 7254	DoJo	4.9	N
UWGM 7255	DoJo	5.8	N
UWGM 7256	Serendipity	2.6	Y
UWGM 7257	DoJo	7.6	Y
UWGM 7258	DoJo	5.5	N
UWGM 7259	DoJo	3.6	N
UWGM 7260	DoJo	6.5	N
UWGM 7261	DoJo	5.3	Y
UWGM 7264	Serendipity	7.52	Y
UWGM 7270	Serendipity	5.1	Y
UWGM 7408	DoJo	4	Y
UWGM 7409	DoJo	4.8	Y
UWGM 7410	DoJo	4.4	Y
UWGM 7411	DoJo	5	Y
UWGM 7412	Serendipity	4.3	Y
UWGM 7413	DoJo	4.4	Y
UWGM 7414	DoJo	6	Y
UWGM 7415	Serendipity	5.9	Y
UWGM 7416	Serendipity	9.2	Y
UWGM 7417	Serendipity	5.1	Y
UWGM 7418	DoJo	7.9	Y
UWGM 7419	DoJo	6.2	Y
UWGM 7421	DoJo	9.8	Y
UWGM 7422	Serendipity	5.2	Y
UWGM 7423	DoJo	7.4	Y
UWGM 7424	Serendipity	4.8	Y
UWGM 7425	DoJo	6.9	N
UWGM 7426	Serendipity	3.4	Y
UWGM 7427	Serendipity	4	Y
UWGM 7429	DoJo	3.2	Y
UWGM 7430	DoJo	5.1	Y
UWGM 7431	DoJo	11	Y
UWGM 7432	DoJo	6.7	Y
UWGM 7436	DoJo	2.8	N
UWGM 7437	Independence	5.7	Y
UWGM 7438	Serendipity	7.7	Y
UWGM 7439	Serendipity	5.6	N
UWGM 7440	Independence	3.3	Y
UWGM 7441	DoJo	6.4	Y
UWGM 7442	DoJo	4.1	Y
UWGM 7443	DoJo	8.5	Y
UWGM 7444	DoJo	7.3	Y

### (b) Systematic palaeontology

Tetrapoda Jaekel, 1909

Temnospondyli Zittel, 1888

Stereospondyli Zittel, 1887

Genus *Ninumbeehan* gen. nov.

**Figure 3.**
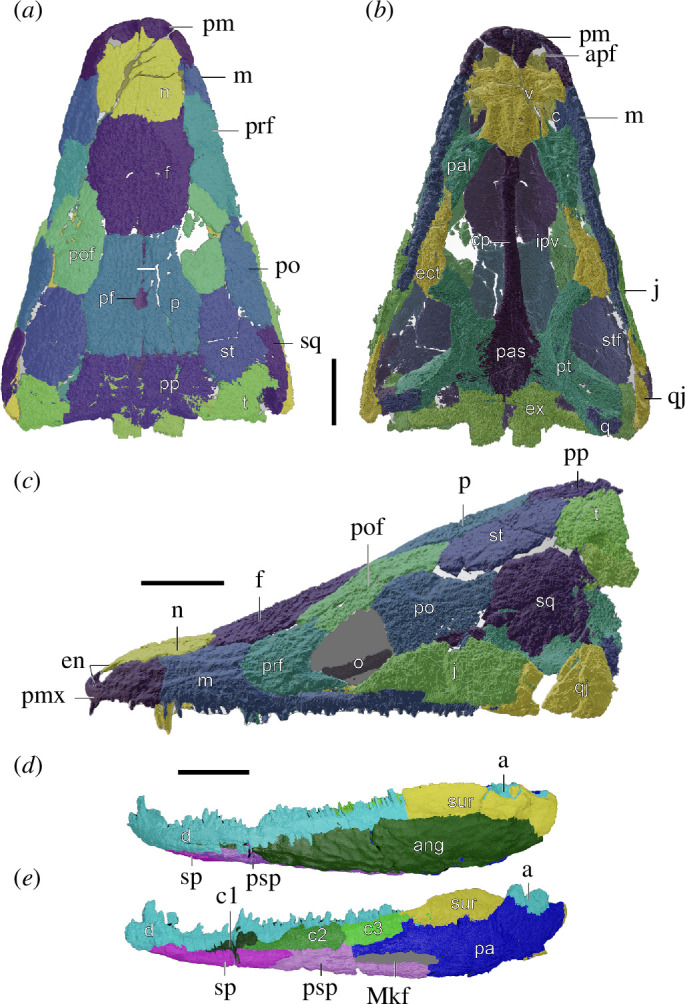
Reconstruction of cranium and lower jaw of *Ninumbeehan dookoodukah* based on UWGM 7264 and UWGM 2164, respectively. Holotype cranium (UWGM 7264) in (*a*) dorsal, (*b*) ventral, and (*c*) left lateral views. Paratype mandible (UWGM 2164) in (*d*) labial, and (*e*) lingual views. Abbreviations: a, articular; ang, angular; apf, anterior palatal fenestra; c, choana; c1, coronoid 1 (=’precoronoid’); c2, coronoid 2 (=’intercoronoid’); c3, coronoid 3 (=’coronoid’); cp, cultriform process of the parasphenoid; d, dentary; ect, ectopterygoid; ex, exoccipital; f, frontal; ipv, interpterygoid vacuity; j, jugal; Mkf, Meckelian fenestra; m, maxilla; n, nasal; en, external naris; o, orbit; p, parietal; pa, prearticular; pal, palatine; pas, parasphenoid; pf, parietal foramen; pm, premaxilla; po, postorbital; pof, postfrontal; pp, postparietal; pt, pterygoid; q, quadrate; qj, quadratojugal; sp, splenial; psp, postsplenial; sq, squamosal; st, supratemporal; stf, subtemporal fenestra; sur, surangular; t, tabular; v, vomer. All scale bars equal to 1 cm.

*Type species*. *Ninumbeehan dookoodukah* gen. et sp. nov. (figure 3; electronic supplementary material, figures S3 and S8−S11).

*Etymology*. The name results from a collaborative partnership between the authors of this publication and the seventh-grade students from the Fort Washakie School (see electronic supplementary material, audio S1, for the pronunciation and historical context by R.T.). From the Shoshone language, *Ninumbee* is the name for the mountain-dwelling Little People who hold an important place in Shoshone culture (among others), -*han* is the possessive affix indicating an affiliation with the *Ninumbee*, *dookoo* means ‘flesh’ and *dukah* means ‘eater’. Altogether, *Ninumbeehan dookoodukah* means ‘Little People’s flesh eater’, honouring the Little People and referencing the sharp teeth of the fossil (urn:lsid:zoobank.org:pub:092A6582-4988-4A57-B695-17557CA516A4). Our intent is to pay tribute to the Eastern Shoshone people, their language and the land to which they belong.

*Locality and horizon*. The Serendipity beds are a locally exposed laterally extensive (km scale) paedogenically modified fluvial sandstone in the upper 10 m of the Jelm Formation near the town of Dubois, Fremont County, Wyoming, USA, on land managed by the Bureau of Land Management (excavation permit: PA16-WY-251; surface permit: PA16-WY-251). The type specimen, UWGM 7264, was recovered from the Serendipity site in a largely complete burrow surrounded by host rock (see the electronic supplementary material for more details).

*Differential diagnosis*. Diminutive stereospondyl sharing characteristics with *Chinlestegophis*, *Rileymillerus* and *Almasaurus*. Autapomorphies include a ventrally sloping unornamented posterior process along the midline formed by the postparietals and anterior dorsal ribs subequal in length to the dorsoventral height of the skull and mandible. Differentiated from *Almasaurus* and tentatively differentiated from *Rileymillerus* (see the electronic supplementary material on interpretations of *Rileymillerus*) by the lack of a lacrimal. Differentiated from *Chinlestegophis* and *Almasaurus* by an expanded posterolateral portion of the premaxilla at the lateral margin of the naris. Differentiated from *Chinlestegophis* and *Rileymillerus* by the lack of a palatine contribution to the skull roof. Further differentiated from *Almasaurus* by a wider posterior portion of the parietal with lateral sutures oriented parallel to the midline, a cultriform process of the parasphenoid that is ventrally flat throughout its length, a palatoquadrate fissure and an expanded posterolateral portion of the premaxilla at the lateral margin of the naris. Further differentiated from *Rileymillerus* by the presence of a falciform crest of the squamosal, pineal foramen in posterior half of parietal, presence of otic notch and presence of tabular horn. Further differentiated from *Chinlestegophis* by a smaller, dorsal otic notch, narrower cultriform process of the parasphenoid and supratemporal subrectangular (as opposed to subcircular).

*Ninumbeehan dookoodukah* gen. et sp. nov.

*Diagnosis.* As for genus.

*Holotype*. UWGM 7264, a well-preserved partial anterior skeleton including the skull, mandibles and pectoral girdle elements in articulation (figure 3; electronic supplementary material, figures S3 and S8−S11). The left humerus, radius, ulna and partial manus are preserved in articulation across the left clavicle. Several ribs are loosely articulated, and several intercentra are disarticulated but clearly associated. Additional postcranial elements are preserved within the matrix but are not prepared, and their boundaries are unclear from micro-computed tomography (μCT) scans.

*Paratypes*. UWGM 2164, a slightly deformed complete skull preserved with left and right mandibular rami, pectoral girdle and disarticulated postcrania (electronic supplementary material, figure S4); UWGM 5856, a skull with mandibles, a disarticulated rostrum and right ‘cheek’ and several postcranial elements including ribs and intercentra (electronic supplementary material, figures S5, S7 and S8); UWGM 7040, partial skull with right lateral side and jaw preserved and an articulated pectoral girdle (electronic supplementary material, figure S6).

*Description*. The cranial anatomy of *Ninumbeehan* is well-represented across the holotype and paratypes, allowing for a comprehensive description (electronic supplementary material, text S1). The largest skull of *Ninumbeehan* is significantly smaller than most other Triassic stereospondyls although larger than the ‘latiscopids’ *Chinlestegophis* and *Rileymillerus*, with the largest known skulls reaching approximately 7 cm long (figure 3). The components of the skull roof are consistent with other stereospondyls, though it lacks a lacrimal. The skull of *Ninumbeehan* is wedge-shaped in lateral view, a morphology that might have played a role in burrow construction. Lateral line sulci incise the skull roof, which is considered indicative of a predominantly aquatic life history owing to desiccation-induced necrosis [24]. *Ninumbeehan* has a small, well-defined, posterolaterally open otic notch (figure 3*a,c*) and long stapes indicative of a tympanic ear (electronic supplementary material, figure S7) [25,26].

The occipital margin of the skull is mostly straight, with the exception of a posterior projection on the midline suture of the postparietal that is ventrally offset by a sloping margin. The well-developed falciform crest is medially offset from the body of the squamosal by a stepped and unornamented margin (electronic supplementary material, figure S8). It may have served as an attachment site for epaxial musculature to stabilize the head during burrow excavation [27]. The mandibular ramus of *Ninumbeehan* bears a secondary tooth row formed by the complete coronoid series lingual to the marginal dentition of the dentary (figure 3*e*), similar to that observed in *Chinlestegophis* and caecilians, though the identity of the elements bearing the lingual row of teeth in caecilians is disputed [28]. All teeth are monocuspid and non-pedicellate. The posterior lingual dentition is subequal in size and parallel to the marginal dentition but decreases in size mesially. The lingual dentition is level to the marginal dentition, contributing equally to occlusion. Additionally, *Ninumbeehan* possesses large dentary and palatal fangs relative to the marginal dentition. The combination of an additional row of teeth participating in occlusion and retention of large fangs may offer a more advantageous grasp for prey capture.

Unique among stereospondyls, the anterior dorsal ribs of *Ninumbeehan* are elongated. The length of one rib is equivalent to over 90% of the dorsoventral height of the combined vaulted skull and mandible. However, it was unlikely that the ribs were entirely vertically oriented in life, given the presence of a well-ossified pectoral girdle [29]. The function of the elongated ribs is unclear, but terrestrial temnospondyls such as dissorophoids have elongated ribs that supported musculature for respiration on land. The ribs throughout the trunk are slightly curved and lack uncinate processes like those of *Almasaurus* [30] and *Chinlestegophis* [18].

The forelimb of *Ninumbeehan* is short and lacks processes for any significant musculature (electronic supplementary material, figures S8 and S9), and the length is approximately one-third the length of the skull, limiting the animal to head-based burrowing. The preserved elements of the forelimb (humerus, radius, ulna and metacarpals) are subequal in length (table 2; electronic supplementary material, figures S9 and S10). The pelvic girdle is currently unknown.

**Table 2.** Measurements of the holotype and paratypes of Ninumbeehan dookoodukah gen. et sp. nov. Measurements of the skull roof and their abbreviations largely follow Sulej (2007). Exact positions for measurements are included in electronic supplementary material, figure S2. Missing values are denoted by a hyphen ( - ), and underlined values were doubled to account for missing or distorted bilaterally symmetrical features. *The skull roof of UWGM 2164 is slightly dorsoventrally crushed. **The ventral margin of the orbit of UWGM 7040 is slightly distorted. ***This measurement represents a minimum length of the rib because the distal end is not visible in UWGM 2164.

Catalog Number	UWGM 7264	UWGM 2164	UWGM 5856	UWGM 7040
Midline skull roof length (ML)	67.3	51	-	52.1
Prepineal skull length (PRL)	46.5	38.6	-	37.8
Postpineal skull length (PSL)	18.5	12.3	14.8	13.9
Postnarial skull width (PNW)	14.7	12	-	13.9
Skull width at anterior orbit (POW)	26.4	27.3	23.8	28
Skull width at posterior orbit (PSW)	35	31.2	28.6	34
Skull roof to ventral side of mandible height (SMH)	37.7	27.7*	-	27.2
Intraorbital width (IOW)	26.5	21.5	20.4	23
Orbit length (OL)	10	9.1	-	10.3**
Orbit height (OH)	9.5	8	-	9.2
Intranotch width (between otic notches) (INW)	39.1	27.8	29	29.8
Maximum skull width (MSW)	44.7	39.1	-	39.2
Clavicle length (CLL)	35.8	-	-	26.8
Clavicle maximum width (CMW)	16.5	11.4	-	10.8
Humerus length (HL)	9.2	-	-	8.5
Humerus distal end max. width (HDW)	7.9	-	-	-
Radius length (RAL)	7	-	-	-
Ulna length (ULL)	5.8	-	-	-
Metacarpal length (may be incomplete) (MCL)	6.4	-	-	-
One anterior trunk rib length (RL)	35	28.7***	-	-

### (c) Phylogenetic interpretation

**Figure 4.**
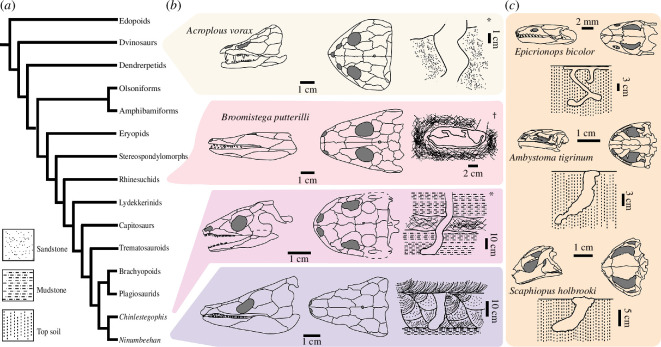
Phylogenetic distribution of known cases of burrowing and burrow morphology among temnospondyls, including modern amphibians. (*a*) Simplified phylogeny of temnospondyls. (*b*) Cranial reconstructions of fossil temnospondyl taxa in lateral (left) and dorsal (centre) view with schematic drawings of associated burrow morphology (right). (*c*) Cranial reconstructions of extant amphibians in lateral (left) and dorsal (right) view with schematics of burrow morphology (below). Asterisks denote burrows that were found in proximity to temnospondyl body fossils. The dagger denotes the secondary occupancy of a burrow.

We conducted phylogenetic analyses of temnospondyls, excluding lissamphibians, using a recently published dataset as our base [28]. The final matrix consisted of 49 taxa and 296 morphological characters. Our analyses recover *Ninumbeehan* as a member of a ‘latiscopid’ clade of diminutive stereospondyls, including *Almasaurus*, *Chinlestegophis* and *Rileymillerus* (electronic supplementary material, figures S12 and S13). A total of 32 most parsimonious trees were recovered with 1261 steps (retention index = 0.293; consistency index = 0.637). Under Bayesian inference, *Ninumbeehan* was recovered as the sister taxon of *Almasaurus* forming a clade that is sister to a clade composed of *Chinlestegophis* and *Rileymillerus* (electronic supplementary material, figures S12 and S13). We find that ‘latiscopids’ cover a range of skull morphology, from the rounded, parabolic skulls of *Chinlestegophis* to the triangular skulls of *Ninumbeehan*, demonstrating burrowing capabilities across varying cranial shapes. Our results underscore the widespread evolution of behavioural response to seasonal extremes and provide further support for fossoriality in other ‘latiscopid’ stereospondyls (figure 4; [18]).

## 3. Discussion

**Figure 5.**
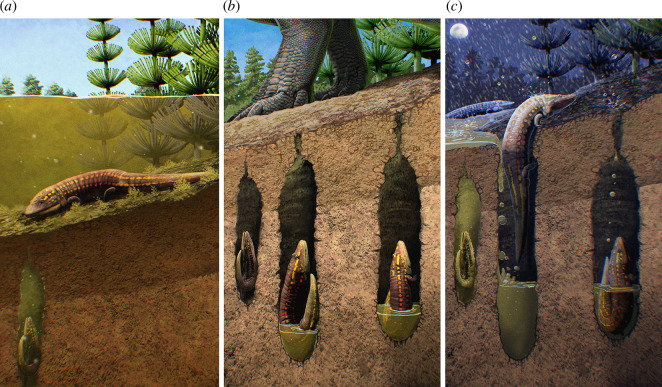
Life reconstruction of *Ninumbeehan dookoodukah* in a palaeoenvironment reconstruction depicting its life and burrowing across seasons. (*a*) *Ninumbeehan* is resting on the edge of a river with a low water table as the wet season ends. (*b*) A small community of *Ninumbeehan* are in their estivation burrows at the apex of the arid season. (*c*) The onset of the humid season brings rain, triggering a mass emergence of *Ninumbeehan* from their estivation burrows. Some individuals expire before the incoming rains, leaving them to be preserved as fossils. Copyright: Gabriel N. Ugueto.

Our discovery of abundant tetrapod burrows in the upper Jelm Formation represents, to our knowledge, the first unambiguous evidence of vertebrate behavioural adaptation to extreme seasonality near the early-Late Triassic palaeoequator (see figure 5 for life reconstruction). The recovery of abundant well-preserved and articulated remains of *Ninumbeehan* has revealed aspects of the anatomy and ecology of the enigmatic ‘latiscopid’ temnospondyls (e.g. *Chinlestegophis*, *Rileymillerus* and *Latiscopus*) [18,31,32]. As with other ‘latiscopids’, *Ninumbeehan* possesses a narrow, wedge-shaped skull and small, laterally directed orbits reminiscent of modern burrowing and estivating amphibians (e.g. *Siren* and *Amphiuma*) [33] and consistent with adaptation for headfirst sediment compaction during burrow construction. *Ninumbeehan* appear to have burrowed into the river channel during seasonal desiccation into underlying point bar deposits from previous cycles of river avulsion, and the beds probably represent a time-averaged assemblage of decades to centuries of seasonal cycles. Seasonal estivation in *Ninumbeehan* is consistent with other reports of temnospondyl–burrow associations [18]. However, *Ninumbeehan* are the first known unambiguous Triassic temnospondyls capable of burrow excavation rather than opportunistic occupation of burrows produced by other animals. These observations highlight the dynamic nature of palaeoequatorial Triassic environments illustrating how some tetrapods navigated behavioural strategies to mitigate harsh climates.

Given the stratigraphic position of the burrow horizon, the Serendipity beds represent the oldest fossiliferous Late Triassic terrestrial strata of the western USA and may precede the CPE (*ca* 234−232 Ma) [7]. Prior to and after the CPE, much of the climate on the continent would have been driven by monsoons, causing alternating seasons of humidity and aridity across the Triassic palaeoequator [4]. Although laterally accreted sediment alternating with mudstone beds is consistent with meandering rivers in a floodplain environment, the presence of periodic estivation horizons in association with pedogenic carbonate and mud cracks indicates that this river system was strongly seasonally influenced and may have been seasonally dry, corresponding to a monsoon-driven climate.

Previous workers have attributed the paucity of vertebrate body fossils in the Carnian of North America to inhospitable conditions limiting vertebrate presence despite seasonal increases in humidity [34]. We show that this is not the case and that vertebrates, including aquatic or semiaquatic temnospondyls, were not only present but locally abundant in this harsh environment. Their survival is linked to specific adaptations to seasonal aridity, notably burrow excavation and estivation, in a manner similar to vertebrate adaptations observed within modern monsoonal systems [35]. Although prior workers have pointed to amniote-specific adaptations (e.g. the amniotic egg and physiological pathways for concentrating urine [36]), modern amphibians primarily mitigate desiccation via a combination of sheltering and seasonal torpor [37]. The clear use of these strategies by *Ninumbeehan* demonstrates that the megamonsoon did impose strong selective pressure on vertebrate communities, but it did not systematically exclude major taxa such as temnospondyls. Previously identified scarcity of body fossils and reduced taxonomic diversity may be a consequence of limited sampling in this poorly studied interval rather than a real palaeobiological signal [9].

Our finding of extensive seasonal estivation in a Triassic temnospondyl hints at the importance of seasonality in shaping the early stages of amphibian evolution. Modern amphibians employ a plethora of ecological and life-history strategies for surviving and thriving in ephemeral wetland systems, including burrowing [37]. Many of these strategies, such as metamorphosis [38] and polyphenism [39], appear to have originated relatively early in amphibian evolution, potentially in response to the increasing severity of the megamonsoon of the Late Palaeozoic. Estivation is an important adaptation for terrestrial and aquatic vertebrates living in modern monsoon systems [37] and extends at least as far back as the onset of the megamonsoon in the Early Permian [40] and possibly as early as the latest Devonian [41]. Estivation does not require headfirst burrowing, and modern estivating amphibians employ a range of strategies to find or excavate estivation chambers, including sheltering in existing crevices, forelimb or hindlimb-driven burrow excavation or commensal relationships with other burrow-producers [37]. It is unlikely that estivation evolved uniquely within ‘latiscopids’; rather, it is likely that burrow excavation in ‘latiscopids’ is an extrapolation of estivation behaviours more broadly distributed across temnospondyls (e.g. estivation in secondarily occupied burrows) [15]. We tentatively propose that estivation may have been widespread among temnospondyls and that specialized head-assisted burrowing adaptations may have arisen in certain lineages, such as ‘latiscopids’, as a direct adaptation to increased seasonality across Pangaea. This may explain the potential endemism of ‘latiscopids’ within the northern hemisphere of the Triassic.

It is, thus, worth considering whether estivation may be partially responsible for larger patterns of temnospondyl adaptation and diversification. Most notably, earliest Triassic temnospondyl faunas are both uniquely diverse and comprised many first occurrences of major temnospondyl clades [42], in parallel with similar trends in lungfishes [43] and other disturbance specialists, suggesting that this group thrived in the harsh earliest Triassic climate in depositional basins such as the Karoo Basin [44]. If so, estivation within burrows by temnospondyls would fit broader patterns in the Triassic recovery faunas; burrowing has been recognized in earliest Triassic therapsids [14] and may have played a role in survivorship across the End-Permian Mass Extinction. Harsh seasonality and common plastic physiological responses across temnospondyl clades might also explain high variability in growth patterns inferred from osteohistology [45]. Given recent work showing that temnospondyls preferentially exploited emerging ephemeral and dryland systems during the early Permian [46], we suggest that temnospondyls may have been more tolerant of disturbed environments than currently recognized. The *Ninumbeehan* estivation horizons we report here serve as a testament to the strong adaptive advantage for temnospondyls (including the forerunners of all three modern lissamphibian orders) as they encountered increasingly harsh seasonal climates through the Permian and Triassic.

## 4. Material and methods

### (a) Phylogenetic analysis

Morphological analyses of temnospondyls, to the exclusion of lissamphibians, were conducted using a matrix based on Kligman *et al*. [28]. Characters for testing lissamphibian relationships among temnospondyls that were added in previous iterations of the matrix (e.g. [18,47]) were removed. All characters were weighted equally and unordered in the maximum parsimony and Bayesian inference analyses. The matrix was analysed in TNT 1.5 [48]. We calculated a strict consensus tree from the most parsimonious trees (electronic supplementary material, figure S12). We analysed the matrix using Bayesian inference under the Mkv model [49] in MrBayes 3.2.7 [50]. We produced a maximum clade credibility tree with posterior probability values mapped onto the interior nodes (electronic supplementary material, figure S13).

### (b) Computed tomography and three-dimensional data

Tomographic analysis of specimen UWGM 2164 was conducted using the Imaging and Medical Beamline at the Australian Nuclear Science and Technology Organisation’s (ANSTO) Australian Synchrotron, Clayton, Victoria, Australia (see the electronic supplementary material for detailed analytical parameters). UWGM 7264 was µCT scanned at the University of Chicago Department of Organismal Biology and Anatomy PaleoCT laboratory (RRID:SCR_024763) on a Phoenix V|tome|x S240 with a dual 180 tube and a 0.7 mm Cu filter (200 kV, 200 µA, voxel size = 76.5 µm). The three-dimensional CT data of fossils were visualized and segmented using Dragonfly (https://www.theobjects.com/dragonfly/). Mesh files were exported from Dragonfly as .stl files and imported to Blender (https://www.blender.org/) for further examination and reconstruction.

## Data Availability

μCT and synchrotron scan data of UWGM 2164 = ID: 000609124 (https://www.morphosource.org/concern/media/000609415); UWGM 7264 = ID: 000609133 (https://www.morphosource.org/concern/media/000609133); UWGM 5856 = ID: 000609139 (https://www.morphosource.org/concern/media/000609139) are available on Morphosource.org. Code for TNT and MrBayes scripts used in the phylogenetic analyses conducted herein are available in Zenodo [51]. The matrix is available for download under project 5115 on Morphobank (http://dx.doi.org/10.7934/P5115). Supplementary material is available online [52].
